# Deciphering the mechanism of hydrogen peroxide formation in ultrasound-mediated water-in-oil microdroplets†

**DOI:** 10.1039/d4sc08098j

**Published:** 2025-03-06

**Authors:** Xiaohu Zhou, Shutong Du, Wenjuan Zhang, Bo Zheng

**Affiliations:** a Institute of Chemical Biology, Shenzhen Bay Laboratory Shenzhen 518132 China zhouxh@szbl.ac.cn bozheng@szbl.ac.cn

## Abstract

Microdroplet chemistry has emerged as a fascinating field, demonstrating remarkable reaction acceleration and enabling thermodynamically unfavorable processes. The spontaneous generation of hydrogen peroxide (H_2_O_2_) in water microdroplets presents a particularly intriguing phenomenon with significant implications for green chemistry and prebiotic processes. However, the transient nature of conventional microdroplets has hindered in-depth mechanistic investigations. This study employs ultrasound-mediated water-in-oil microdroplets to elucidate the underlying mechanism of H_2_O_2_ generation. Under ultrasound irradiation, the H_2_O_2_ concentration increases linearly with a production rate of 0.24 mM min^−1^, reaching 14.37 mM after one hour. Notably, 99% of this production occurs at the water–oil interface, corresponding to approximately 0.10 mM m^−2^ min^−1^. Quantification of key intermediates reveals that superoxide radical (·O_2_^−^) concentrations are approximately tenfold higher than those of H_2_O_2_ and thousandfold higher than those of hydroxyl radicals (·OH). Through radical scavenging and isotope labeling experiments, we identify dissolved oxygen as the primary source and ·O_2_^−^ as the main intermediate in H_2_O_2_ formation, following the pathway: O_2_ → ·O_2_^−^ → H_2_O_2_. We validate the critical role of the water–oil interface in initiating H_2_O_2_ production *via* charge separation reactions and demonstrate the significance of proton availability and surface propensity in facilitating efficient H_2_O_2_ generation. These findings not only advance our understanding of microdroplet interfacial chemistry but also offer potential applications in atmospheric chemistry, green disinfection, and origins of life research.

## Abstract 2

Ultrasound-driven water-in-oil microdroplets achieve rapid H_2_O_2_ production (14.37 mM h^−1^) *via* an interfacial O_2_ →·O_2_^−^ → H_2_O_2_ cascade reaction, enabled by synergistic effects of cavitation, enhanced O_2_ availability, and interfacial dynamics.

## Graphical abstract

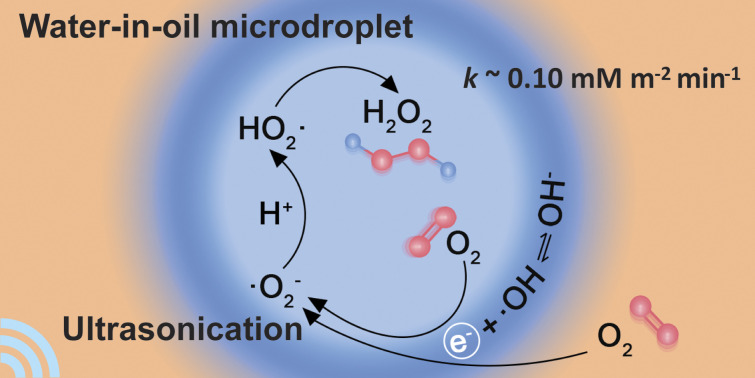

## Introduction

Microdroplet chemistry has garnered considerable attention due to its extraordinary ability to accelerate chemical reactions by two to six orders of magnitude and to drive reactions that typically require catalysts in the bulk phase.^1–9^ These reactions encompass not only simple oxidation/reduction processes^10–12^ but also pivotal synthetic transformations, including C–C, C–N, and C–O bond formation,^13–16^ as well as reactions involving biomolecules and abiotic synthesis.^17–20^ Microdroplet chemistry holds immense potential in fields such as green chemistry, environmental science, prebiotic chemistry, and astrobiology. Despite the consensus that the aqueous interface of microdroplets plays a crucial role in reaction rate acceleration, the detailed mechanisms remain elusive.^1,5^ Unlike bulk solvation, the theoretical understanding of interfacial solvation is still in its infancy.^5^ Given the ubiquity of water, comprising 71% of the Earth's surface and more than half of every living cell, elucidating the mechanisms of microdroplet chemistry is both fundamentally important and practically relevant.^21^

One of the most debated phenomena in microdroplet chemistry is the spontaneous formation of hydrogen peroxide (H_2_O_2_) in pure water microdroplets smaller than 10 μm.^22–28^ Zare and colleagues first reported that sprayed water microdroplets could spontaneously generate H_2_O_2_,^22^ a finding later extended to condensed water microdroplets.^23^ The yield of H_2_O_2_ is influenced by factors, such as microdroplet size, with smaller droplets achieving higher concentrations,^22–24,26^ and environmental conditions, including the relative humidity^29^ and substrate temperature.^30^ These findings have sparked interest in microdroplet interfaces as potential platforms for catalyst-free H_2_O_2_ production.

Despite significant progress, the exact mechanism underlying H_2_O_2_ formation in microdroplets remains not fully understood.^31–34^ Based on their findings, Zare and colleagues proposed that the primary mechanism involves a strong electric field at the air–water interface^31^ that facilitates charge separation, converting hydroxide ions (OH^−^) into hydroxyl radicals (·OH), which then recombine to form H_2_O_2_.^22–25,32^ Additionally, apart from the ·OH radical recombination, George and co-workers proposed a second reaction pathway to form H_2_O_2_ in the presence of oxygen.^26,27^ In this pathway, dissolved oxygen reacts with the solvated electrons, forming superoxide radicals (·O_2_^−^), which subsequently react with hydrogen ions (H^+^) to form hydroperoxyl radicals 
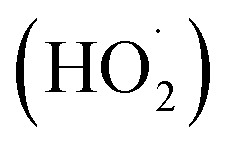
 that self-recombine to form H_2_O_2_. Recent theoretical studies have supported this mechanism by revealing that an increased amount of hydroxide dissociates at interfaces due to reduced solvation.^35,36^ Moreover, the detected presence of ·OH radicals in microdroplets lends further credence to this hypothesis.^24,26,37^ In addition, Colussi proposed an alternative mechanism that involves collisions between oppositely charged microdroplets to produce H_2_O_2_.^33^

In contrast, Mishra and colleagues during rigorous studies have contested the concept of spontaneous H_2_O_2_ formation at the air–water interface,^38^ arguing that the observed H_2_O_2_ could arise from experimental artifacts such as ambient ozone contamination^39^ or water–solid interface effects.^40^ These contradictions are reinforced by Williams and co-workers' recent observation that hydroxyl radicals are not spontaneously generated in inactivated water droplets,^41^ paired with theoretical evidence showing that the electric fields at the air–water interface are insufficient to induce spontaneous electron transfer.^42,43^ Together, these findings challenge the hypothesis of spontaneous H_2_O_2_ generation at the air–water interface. These findings underscore the necessity for stringent experimental controls and reveal the intricate nature of interfacial chemistry in microdroplets.

However, the transient nature of microdroplets, which exist for mere milliseconds in the case of sprayed microdroplets or several minutes for condensed microdroplets,^22,23^ and the relatively low yield of H_2_O_2_ (<30 μM)^44^ pose significant challenges to further in-depth investigation into the underlying mechanisms. Elucidating the primary source of H_2_O_2_ formation and gaining a quantitative understanding of the interplay between various reactive oxygen species (H_2_O_2_, ·OH, and ·O_2_^−^) during this process are crucial for advancing our knowledge in this burgeoning field.

Recently, Lee *et al.* introduced an innovative approach using ultrasound-mediated water microdroplets with extended lifetimes, ranging from milliseconds to hours, by employing an oil–water interface instead of an air–water interface to create the microdroplets.^45^ The study demonstrated that the oil-confined aqueous microdroplets continuously generated hydroxyl radicals near the interface, resulting in H_2_O_2_ formation at mM concentrations, enabling the synthesis of polymers at high reactant concentrations ranging from mM to M. However, this work primarily focused on applying this setup for radical polymerization in polymer synthesis, without delving into the underlying mechanism of H_2_O_2_ formation.

In this study, we aimed to elucidate the underlying mechanism of H_2_O_2_ generation using ultrasound-mediated water-in-oil microdroplets. It should be emphasized that our work focuses strictly on the ultrasound-mediated process rather than on spontaneous H_2_O_2_ formation in the absence of external stimuli. We demonstrated that under ultrasound irradiation, the H_2_O_2_ concentration increases linearly with time, with a production rate of approximately 0.24 mM min^−1^, reaching up to 14.37 mM after 1 hour of irradiation. Notably, 99% of this production occurs at the water–oil interface, corresponding to a surface-area-normalized production rate of approximately 0.10 mM m^−2^ min^−1^, attributed to the combined effects of the water–oil interface, ultrasonic cavitation, and the enhanced solubility and mass transfer rate of O_2_ in oil. We identified and quantified key intermediate radicals during H_2_O_2_ production, finding that concentrations of superoxide radicals (·O_2_^−^) and hydroxyl radicals (·OH) also increased linearly with irradiation time, similar to H_2_O_2_. Notably, the yield of superoxide radicals was nearly 10 times higher than that of H_2_O_2_ and approximately 1000 times higher than that of hydroxyl radicals. Subsequently, we confirmed that the dissolved oxygen is the primary source, and the ·O_2_^−^ serves as the primary intermediate for H_2_O_2_ formation through the radical scavenging and isotope labeling experiments, identifying the reaction pathway: O_2_ → ·O_2_^−^ → H_2_O_2_. Additionally, we validated the essential role of the water–oil interface in initiating H_2_O_2_ production through the charge separation reactions. Lastly, we validated the crucial roles of proton availability and surface propensity in facilitating efficient H_2_O_2_ generation by examining the effects of pH and ionic environments on the aqueous phases. Although this study focuses on ultrasound-mediated H_2_O_2_ formation, which operates under different conditions compared to spontaneous H_2_O_2_ generation in microdroplets, we hope the findings of this study can provide valuable insights for spontaneous H_2_O_2_ generation in microdroplets. This study not only sheds light on the unique physicochemical properties of microdroplets but also has potential implications for atmospheric chemistry, green disinfection, and understanding the origins of life on Earth.

## Results and discussion

### Generation of H_2_O_2_ in ultrasound-mediated water-in-oil microdroplets

**Fig. 1:**
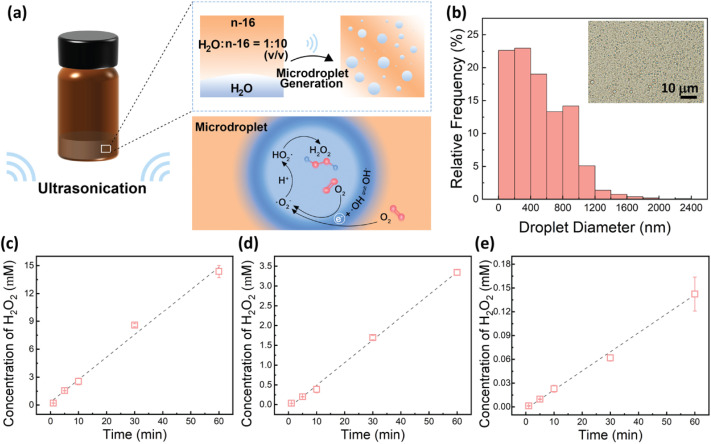
Generation of H_2_O_2_ in ultrasound-mediated water-in-oil microdroplets. (a) Schematic representation of the experimental setup and overall reaction process. (b) Diameter distribution of microdroplets formed by ultrasonic emulsification of a 1 : 10 (v/v) water-to-hexadecane mixture. The inset displays water microdroplets after 5 minutes of ultrasound irradiation. (c) H_2_O_2_ concentration in microdroplets as a function of ultrasound irradiation time. (d) H_2_O_2_ concentration in microdroplets following partial removal of dissolved O_2_ by N_2_ purging, as a function of ultrasound irradiation time. (e) H_2_O_2_ concentration in 2.2 mL DI water as a function of ultrasound irradiation time.

We first investigated the generation of H_2_O_2_ in ultrasound-mediated water-in-oil microdroplets. The experimental setup and the proposed reaction pathway are illustrated in Fig. 1a. In our experiment, 200 μL deionized (DI) water was emulsified into microdroplets within 2 mL hexadecane oil using an ultrasonic bath (40 kHz, 200 W). The resulting water microdroplets had an average diameter of 0.5 μm (Fig. 1b).

The concentration of H_2_O_2_ was quantified *via* UV-vis spectroscopy of the aqueous phase collected by centrifugation after ultrasound irradiation (Fig. S1†).^45,46^ As shown in Fig. 1c, the concentration of H_2_O_2_ increased linearly with ultrasound irradiation time, with a production rate of approximately 0.24 mM min^−1^, reaching up to 14.37 mM after 1 hour of irradiation. This finding is consistent with previous reports of H_2_O_2_ production in ultrasound-mediated microdroplets^45^ and significantly exceeds the yields observed in sprayed or condensed microdroplets.^22–24^ The enhanced yield under these conditions could be attributed to the longer reaction time and the effects of ultrasonic cavitation.

We further evaluated the production of H_2_O_2_ in water-in-hexadecane microdroplets after removing dissolved O_2_ by purging with N_2_ for 15 minutes and replacing the vial lid with a N_2_ balloon during ultrasound exposure. It should be noted that this method only partially removed dissolved O_2_ from the liquid phases.^47^ Even with the reduced concentration of dissolved O_2_, the H_2_O_2_ concentration continued to increase linearly with irradiation time, achieving a production rate of 0.057 mM min^−1^ and a yield of 3.34 mM after 1 hour, approximately 23% of the yield obtained without O_2_ removal (Fig. 1d). These findings indicate that dissolved O_2_ may be a major contributor to H_2_O_2_ production.

In contrast with the previous results,^45^ we found that bulk water subjected to the same ultrasound irradiation conditions also generated detectable levels of H_2_O_2_.^48^ Note that due to the experimental setup and ultrasonic bath power, H_2_O_2_ yield is volume-dependent (Fig. S2†). To ensure comparability across results, all samples were maintained at a constant total volume of 2.2 mL. The concentration of H_2_O_2_ in bulk water increased linearly with ultrasound exposure, at a production rate of about 0.0024 mM min^−1^, resulting in 0.14 mM H_2_O_2_ after 1 hour—only 1% of the yield obtained in microdroplets (Fig. 1e). This suggests that ultrasonic cavitation may contribute to H_2_O_2_ formation in bulk water.^23,38^

**Fig. 2:**
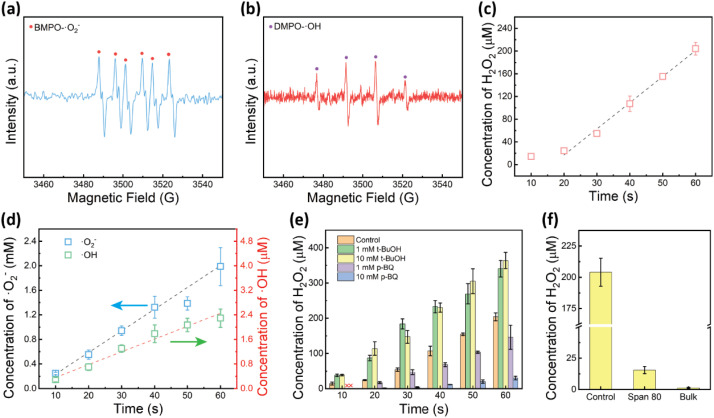
Characterization and quantification of reactive oxygen species in ultrasound-mediated water-in-oil microdroplets. (a and b) EPR spectra of (a) BMPO-·O_2_^−^, (b) DMPO-·OH after 5 minutes of ultrasound irradiation. (c) Quantification of H_2_O_2_ in water microdroplets under ultrasound irradiation for 60 seconds in an air atmosphere. (d) Quantification of ·O_2_^−^ with NBT and ·OH using TA in water microdroplets under ultrasound irradiation within 60 seconds in an air atmosphere. (e) H_2_O_2_ evolution in the presence of various radical scavengers at different concentrations. (f) Comparison of H_2_O_2_ yields in water-in-oil microdroplets with or without a surfactant and in bulk water after 60 seconds of ultrasound irradiation.

To elucidate the contribution of the oil phase to the high yield of H_2_O_2_ production in ultrasound-mediated water microdroplets, we compared the yields of H_2_O_2_ production in two-phase systems with varying ratios of DI water and hexadecane (Fig. S3†). Strikingly, the yield of H_2_O_2_ increased proportionally with the oil-to-water ratio, likely attributable to the enhanced solubility and accelerated mass transfer rate of O_2_ in hexadecane,^49,50^ since the dissolved O_2_ may be a major contributor to H_2_O_2_ production (Fig. 1c and d). However, given the substantial reduction in H_2_O_2_ yield upon interfacial blocking with surfactants (Fig. 2f), coupled with the negligible solubility of H_2_O_2_ in hexadecane,^51^ and since single-phase bulk water produced only 1% of the H_2_O_2_ yield obtained in microdroplets, we may infer that the remaining 99% of H_2_O_2_ formed at the water–oil interface. Utilizing the average microdroplet dimensions (Fig. 1b), we estimated the cumulative water–oil interfacial area to be approximately 2.40 m^2^, resulting in a surface-area-normalized H_2_O_2_ production rate of approximately 0.10 mM m^−2^ min^−1^. This rate is five orders of magnitude higher than the previously reported value of 7.7 nM m^−2^ min^−1^ for static microdroplets in oil.^28^ This substantial increase in the production rate is likely due to the combined effects of dynamic interfacial renewal, ultrasonic cavitation and accelerated mass transfer rates under irradiation.

Previous studies suggested that hydroxyl radicals (·OH) generated from hydroxide anions at the water–oil interface are the primary source of H_2_O_2_, with sufficient radical concentration initiating free radical polymerization.^45^ The studies imply that water could be the main source of H_2_O_2_ formation, following the reaction pathway: H_2_O → ·OH → H_2_O_2_. However, when we attempted to induce microdroplet-mediated radical polymerization using the acrylamide monomer, the subsequent ^1^H NMR analysis showed no detectable polymerization after 1 hour of ultrasound irradiation (Fig. S4†). Our observation suggests that the hydroxyl radicals produced during H_2_O_2_ formation are insufficient to initiate radical polymerization under these conditions.

### Identification and quantification of key intermediates in H_2_O_2_ formation

To elucidate the mechanism of H_2_O_2_ production in the water–oil sonication system, we systematically investigated the intermediates involved in this process. Electron paramagnetic resonance (EPR) spectroscopy was employed to identify the intermediate products and elucidate the reaction pathway of H_2_O_2_ production. For this purpose, 200 μL 100 mM BMPO and 200 μL 100 mM DMPO were employed as the aqueous phase to detect superoxide radicals (·O_2_^−^) and hydroxyl radicals (·OH), respectively.^46,52^

As shown in Fig. 2a and b, after 5 minutes of ultrasound irradiation, the BMPO test exhibited characteristic sextuplet peaks indicative of BMPO-·O_2_^−^ (Fig. 2a), which arises from the reduction of O_2_. Similarly, the DMPO test displayed characteristic quadruplet peaks for DMPO-·OH (Fig. 2b), suggesting that ·OH was generated during the H_2_O_2_ production, likely due to the influence of the strong electric fields at the water–oil interface^22–24,26,31^ and/or the ultrasonic cavitation.^53,54^ These results confirmed the presence of both superoxide radicals (·O_2_^−^) and hydroxyl radicals (·OH) during ultrasound irradiation, indicating that both the dissolved oxygen and water might serve as the main source for H_2_O_2_ production.

To further investigate the formation mechanism, we quantitatively monitored the intermediate products (·O_2_^−^ and ·OH) during H_2_O_2_ production under ultrasound irradiation. Nitroblue tetrazolium (NBT, 2,2′-di-*p*-nitrophenyl-5,5′-diphenyl-(3,3′-dimethoxy)-4,4′-bisphenyleneditetrazolium chloride) was used as the color indicator for the detection and quantification of ·O_2_^−^, while terephthalic acid (TA) was employed to quantify the ·OH.^52^

Upon reduction by ·O_2_^−^, NBT transitions from yellow to blue formazan (Fig. S5†), and the non-fluorescent TA reacts with ·OH to produce fluorescent hydroxyterephthalic acid (hTA) (Fig. S6†). Given the low solubility of NBT and its product, as well as the high yield of H_2_O_2_, our focus was primarily on intermediate products and reaction pathways within the first 60 seconds of ultrasound irradiation (Fig. 2c and d).

We first examined H_2_O_2_ production in water-in-hexadecane microdroplets within 60 seconds of ultrasound irradiation (Fig. 2c). The H_2_O_2_ concentration increased with irradiation time, reaching 204.10 μM H_2_O_2_ after 60 seconds of irradiation. During short irradiation times, the H_2_O_2_ production rate did not exhibit a strong linear fit. However, accounting for the ultrasonic bath's response time and detection limits, excluding the 10-second data point reveals a strong linear correlation between the H_2_O_2_ production rate and irradiation time from 20 to 60 seconds (Fig. 2c). The calculated production rate was approximately 3.40 μM s^−1^ or 0.20 mM min^−1^, which aligns with the previously observed rate of 0.24 mM min^−1^ under 1 hour of irradiation (Fig. 1c). The result confirms a consistent linear relationship between the H_2_O_2_ yield and ultrasound irradiation time across different time scales, suggesting that the underlying reaction mechanism remains constant.

Using the stoichiometric relationship that 1 mole of NBT consumes 2 moles of ·O_2_^−^ (or electrons) to form monoformazan, we determined that the concentration of ·O_2_^−^ increased linearly with the ultrasound irradiation time with the production rate of 0.033 mM s^−1^, which reached 1.98 mM after 60 seconds (Fig. 2d). This was approximately 10 times greater than the yield of H_2_O_2_.

Interestingly, the concentration of ·OH also increased linearly with the ultrasound irradiation time, albeit at a much slower production rate of 0.038 μM s^−1^, yielding only 2.29 μM after 60 seconds (Fig. 2d). This was about 100 times lower than the H_2_O_2_ yield and roughly 1000 times lower than the ·O_2_^−^ concentration, suggesting that ·O_2_^−^ is likely the primary radical intermediate in H_2_O_2_ production.

Furthermore, considering the high reactivity and short lifetimes of ·O_2_^−^ and ·OH, not all radicals were converted to H_2_O_2_, implying that intermediate radicals existed at higher concentrations than the H_2_O_2_ product. These observations reinforce the notion that the oxygen reduction pathway is the main contributor to H_2_O_2_ formation, following the reaction pathway: O_2_ → ·O_2_^−^ → H_2_O_2_.

We extended our investigation to ultrasound irradiation under reduced dissolved O_2_ conditions (Fig. S7†). By N_2_ purging for 15 minutes to remove part of the dissolved O_2_, the H_2_O_2_ production in 60 seconds ultrasound-mediated water-in-oil microdroplets significantly decreased to 33.76 μM, only about 16% of the H_2_O_2_ produced under an air atmosphere (Fig. S7a†). The percentage decrease of the yield was consistent with the results from prolonged irradiation (Fig. 1c and d). Notably, under anaerobic conditions, NBT acted as a direct electron acceptor, forming monoformazan at slightly higher yields with the stoichiometric parameter that 1 mole of NBT consumes 2 moles of electrons (Fig. S7b†).^55^ Interestingly, after partially removing the dissolved O_2_ by N_2_ purging, the amount of ·OH was also markedly reduced under ultrasound irradiation in the N_2_ environment (Fig. S7c†). After 60 seconds of ultrasound irradiation, only approximately 0.30 μM ·OH was produced in a N_2_ atmosphere, about 13% of that observed in air. The decrease in ·OH concentration mirrored the reduction in the H_2_O_2_ yield under a N_2_ environment, suggesting a positive relationship between ·OH levels and the H_2_O_2_ yield, even though their absolute quantities were not comparable.

### Radical scavenging experiments: elucidating the reaction pathway

Next, to further elucidate the mechanism of H_2_O_2_ production, we performed radical scavenging experiments using *p*-benzoquinone (*p*-BQ) and *tert*-butanol (*t*-BuOH) as quenchers for ·O_2_^−^ and ·OH, respectively.^52,56^ Initially, introducing 1 mM *p*-BQ into the aqueous phase resulted in a marked reduction in H_2_O_2_ yield (Fig. 2f). After a brief ultrasound exposure of 10 seconds, H_2_O_2_ was entirely undetectable. Following 60 seconds of ultrasound irradiation, the H_2_O_2_ yield was approximately 71% of the control group. Given the high initial presence of ·O_2_^−^ (Fig. 2d), we increased the *p*-BQ concentration to 10 mM, which led to an 85% decrease in H_2_O_2_ yield after 60 seconds of ultrasound exposure (Fig. 2e). These results strongly indicate that ·O_2_^−^ serves as the primary intermediate for H_2_O_2_ formation, following the reaction pathway: O_2_ → ·O_2_^−^ → H_2_O_2_.

In contrast, the introduction of 1 mM *t*-BuOH as a ·OH quencher significantly enhanced the yield of H_2_O_2_ (Fig. 2e). After 60 seconds of ultrasound irradiation, the H_2_O_2_ yield increased to approximately 170% of the control group. This unexpected outcome suggests that quenching the ·OH radicals promotes H_2_O_2_ production, implying that ·OH is not a direct intermediate in the formation of H_2_O_2_. Furthermore, increasing the *t*-BuOH concentration to 10 mM did not further augment the H_2_O_2_ yield, indicating a saturation effect (Fig. 2e).

To further investigate the effects of the water–oil interface, we introduced the nonionic surfactant Span 80 (1% w/v) into the aqueous phase, which accumulates at the water–oil interface and likely suppresses interfacial reactions by blocking reactive sites. After 60 s of ultrasound irradiation, the presence of the surfactant led to a drastic reduction in the H_2_O_2_ yield, reaching only 15.50 μM, approximately 7% of the H_2_O_2_ yield without the surfactant (Fig. 2f). This substantial decrease confirms the critical role of interfacial effects in H_2_O_2_ production. Notably, the H_2_O_2_ yield with the surfactant remained higher than the yield from bulk water (1.33 μM), possibly due to incomplete interface blockage.

Considering the confirmed presence of ·O_2_^−^ and ·OH radicals (Fig. 2c), the substantial decrease in the H_2_O_2_ yield upon partial removal of dissolved oxygen (Fig. 1c and d), and the marked reduction in the H_2_O_2_ yield upon quenching of ·O_2_^−^ radicals (Fig. 2e) or blocking the interface with a surfactant (Fig. 2f), we propose the following reaction pathway: under the influence of ultrasonic cavitation and a strong electric field at the water–oil interface, hydroxyl radicals (·OH) and solvated electrons (e^−^) are generated through charge separation of hydroxide ions (OH^−^). Dissolved oxygen (O_2_) subsequently accepts these solvated electrons, forming superoxide radicals (·O_2_^−^). These radicals then react with hydrogen ions (H^+^), generating hydroperoxyl radicals 
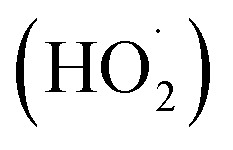
, which subsequently undergo a self-reaction to form H_2_O_2_ (Fig. 1a). This pathway elucidates why ·O_2_^−^ serves as the primary intermediate in H_2_O_2_ production.

The addition of *t*-BuOH, a hydroxyl radical scavenger, shifts the charge separation reaction rightward, leading to increased production of solvated electrons and consequently enhanced the yield of H_2_O_2_. Furthermore, the formation rates of hydroxyl radicals and solvated electrons, constrained by the availability of water–oil interfaces, explain why further increases in *t*-BuOH concentration do not result in additional H_2_O_2_ yield.

To further corroborate the influence of the charge separation reaction, we introduced electron scavengers into the system: 10 mM AgNO_3_ in the aqueous phase and 10 mM CCl_4_ in the oil phase.^46,52,56^ In both cases, the yield of H_2_O_2_ significantly increased (Fig. S8a†). Additionally, the introduction of CCl_4_ as an electron scavenger markedly increased the yield of ·OH radicals (Fig. S8b†), providing direct evidence for the rightward shift of the charge separation reaction. These observations collectively reinforce our proposed mechanism and highlight the critical role of interface dynamics in the H_2_O_2_ production pathway.

### Isotopic labeling techniques for tracing the H_2_O_2_ formation pathway

**Fig. 3:**
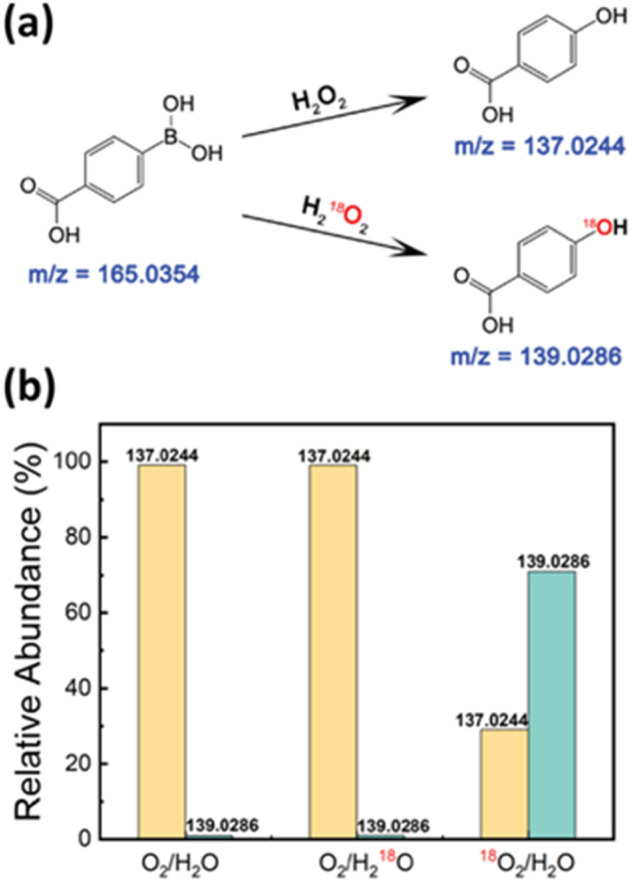
Isotope labeling experiment for elucidating the H_2_O_2_ formation mechanism. (a) Reaction scheme of H_2_O_2_-promoted/H_2_^18^O_2_-promoted deborylation of 4-carboxyphenylboronic acid. (b) Mass spectrometric analysis of the resulting 4-hydroxybenzoic acid.

To further validate the reaction pathway leading to H_2_O_2_ production, we employed oxygen isotope labeling experiments to trace the origin of the oxygen atoms in H_2_O_2_ using mass spectrometry (MS) analysis.^57^ We used 4-carboxyphenylboronic acid as a probe, which reacts with the generated H_2_O_2_ to form 4-hydroxybenzoic acid. If the produced H_2_O_2_ contained the oxygen isotope, the resulting 4-hydroxybenzoic acid would exhibit corresponding isotope signals in the mass spectra (Fig. 3a).

We conducted three sets of experiments to compare the formation of 4-hydroxybenzoic acid: (1) the control experiment with O_2_/H_2_O, (2) a water replacement experiment using O_2_/H_2_^18^O, and (3) an oxygen replacement experiment using ^18^O_2_/H_2_O. The relative intensity of the mass spectrometric peak at 139.02 *m*/*z* in the O_2_/H_2_^18^O setup remained as low as that observed in the O_2_/H_2_O setup. In contrast, the intensity at 139.02 *m*/*z* increased significantly from 1% to 70% in the ^18^O_2_/H_2_O experiment, indicating that the oxygen atoms in the H_2_O_2_ predominantly originated from the dissolved O_2_ (Fig. 3b). It should be noted that despite purging for 15 minutes, we could not completely replace all dissolved O_2_ with ^18^O_2_. These findings further confirm that dissolved O_2_ serves as the primary source of H_2_O_2_ in the reaction pathway.

While the charge separation reaction at the water–oil interface is central to H_2_O_2_ formation (Fig. 2b, e, f, and S6†), our findings raised questions about the minimal recombination of hydroxyl radicals (·OH) into H_2_O_2_ (Fig. 2f and 3b) and the substantially lower levels of ·OH detection compared to H_2_O_2_ and superoxide radicals (·O_2_^−^) (Fig. 2d). Considering the interfacial nature of the charge separation and the prevalence of water and hexadecane in the system, we hypothesized that the highly reactive and short-lived ·OH radicals primarily reacted with hexadecane, resulting in the formation of various organic compounds. This hypothesis was supported by our MS analysis (Fig. S9†).

### Influence of pH and ionic environment on H_2_O_2_ production dynamics

**Fig. 4:**
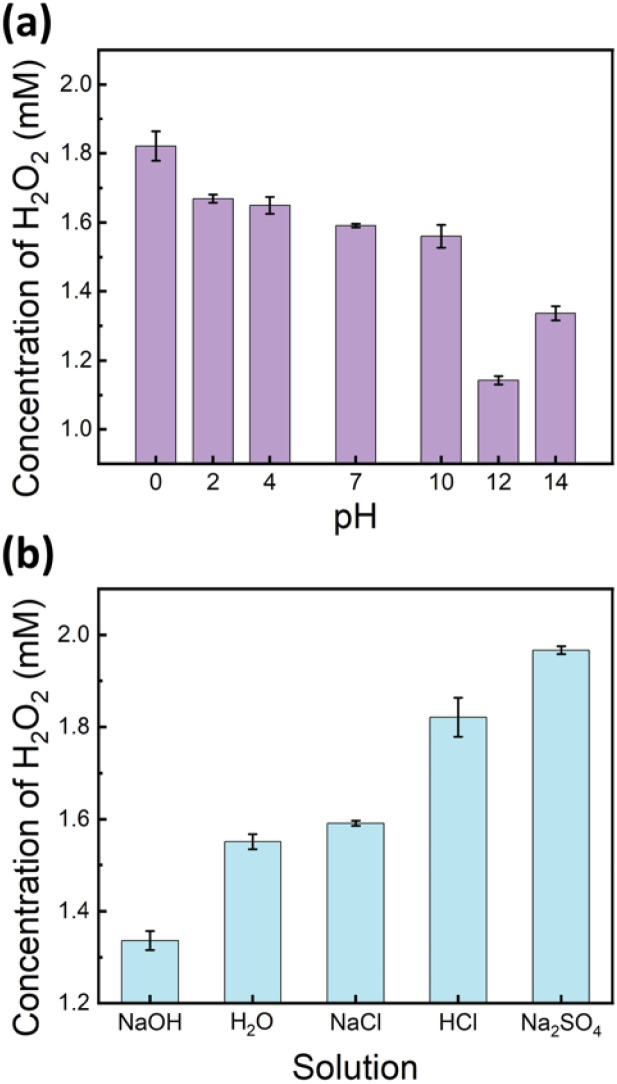
Comparison of H_2_O_2_ yields after 5 minutes of ultrasound irradiation: (a) at different pH levels and (b) in various 1 M salt solutions.

Building on the previous experiments, which identified oxygen oxidation as the primary pathway for H_2_O_2_ production in ultrasound-mediated water-in-oil microdroplets, we hypothesized that lower pH conditions, with an increased concentration of H^+^ ions, would enhance H_2_O_2_ formation. To test this hypothesis, we prepared solutions with pH values ranging from 0 to 14 using the HCl and NaOH solutions and subjected them to ultrasound-mediated reactions. The results demonstrated a positive correlation between the H_2_O_2_ yield and proton concentration ([H^+^]) in the pH range from 0 to 12 (Fig. 4a).

Intriguingly, an unexpected increase in H_2_O_2_ yield was observed at pH 14. This phenomenon may be attributed to altered interfacial dynamics, specifically the adsorption of excess hydroxide ions (OH^−^) at the interface,^58^ which potentially promotes charge separation reactions and consequently enhances H_2_O_2_ production (Fig. 4a). It is noteworthy that the surface tension of water remains relatively constant between pH 1 and 13.^59^ This physicochemical property supports our observation of a consistent positive correlation between the H_2_O_2_ yield and proton concentration ([H^+^]) within this pH range, highlighting the mechanistic relationship between acidity and peroxide formation.

In addition to pH, we investigated the influence of various salts on H_2_O_2_ production. As shown in Fig. 4b, the addition of 1 M NaCl, at a neutral pH, did not significantly affect the H_2_O_2_ yield compared to that of DI water. However, the presence of Na_2_SO_4_, also at neutral pH, significantly enhanced H_2_O_2_ yields, even surpassing those observed in a 1 M HCl solution. The results suggest that beyond the direct effect of proton concentration, SO_4_^2−^ anions with their relatively higher proton transfer efficiency and lower surface propensity^60^ promote charge-separation reactions and hydroperoxyl radical formation, thereby enhancing H_2_O_2_ production. These observations reinforce the critical role of both proton availability and surface propensity in facilitating efficient H_2_O_2_ generation in ultrasound-mediated water microdroplets.

## Conclusions

In this study, we investigated the underlying mechanism of H_2_O_2_ generation in ultrasound-mediated water-in-oil microdroplets. Our investigations revealed a linear increase in H_2_O_2_ concentration under ultrasound irradiation, achieving a remarkable production rate of 0.24 mM min^−1^. After one hour of irradiation, the H_2_O_2_ concentration reached an impressive value of 14.37 mM. Notably, 99% of this yield occurred at the water–oil interface, corresponding to a surface-area-normalized production rate of approximately 0.10 mM m^−2^ min^−1^, arising from synergistic effects, including interfacial dynamics, ultrasonic cavitation, and the enhanced solubility and mass transfer rate of O_2_ in oil.

Through comprehensive radical scavenging and isotope labeling experiments, we identified superoxide radicals (·O_2_^−^) as the principal intermediates in the H_2_O_2_ formation pathway, establishing that dissolved oxygen serves as the primary source. This confirmed the reaction sequence O_2_ → ·O_2_^−^ → H_2_O_2_. Our quantitative analysis further demonstrated that the yield of superoxide radicals was approximately 10 times greater than that of H_2_O_2_, underscoring their pivotal role in the reaction mechanism. Additionally, charge separation reactions at the water–oil interface were found to be integral to H_2_O_2_ formation, highlighting the crucial influence of interfacial dynamics on reaction kinetics in microdroplet systems. Moreover, our investigation into the effects of pH and ionic environments revealed that proton availability and surface propensity significantly affect H_2_O_2_ production, emphasizing the impact of pH and ionic composition on interfacial chemistry.

This study advances the understanding of microdroplet chemistry by providing detailed insights into the generation of H_2_O_2_ and the essential role of interfacial effects. Although this study focuses on ultrasound-mediated H_2_O_2_ formation, which operates under different conditions compared to spontaneous H_2_O_2_ generation in microdroplets, we hope the findings of this study could provide valuable insights for the spontaneous H_2_O_2_ generation in microdroplets. These findings have broader implications for atmospheric chemistry, green disinfection strategies, and prebiotic chemistry, offering avenues for optimizing H_2_O_2_ production and deepening our comprehension of chemical processes at aqueous interfaces.

## Data availability

The data that are discussed in this article are available in the supplementary information of the corresponding articles referenced. Additional details on materials, methods, and experimental results, including calibration curves (Fig. S1, S5 and S6†), H_2_O_2_ yield dependence on the sample volume (Fig. S2†) and water-to-hexadecane ratio (Fig. S3†), ^1^H NMR spectra (Fig. S4†), quantification of H_2_O_2_, monoformazan and ·OH in a N_2_ environment (Fig. S7†), evolution of H_2_O_2_ and ·OH in the presence of electron scavengers (Fig. S8†), and mass spectra (Fig. S9†) are available in the ESI.†

## Author contributions

X. Z. and B. Z. conceived the project. S. D. conducted the experiments. W. Z. helped develop the methodology. X. Z. and S. D. conducted data analysis. X. Z. drafted the original manuscript. X. Z. and B. Z. reviewed and edited the manuscript. B. Z. provided financial support and supervised the work. X. Z. and S. D. contributed equally to this work.

## Conflicts of interest

The authors declare no competing financial interest.

## Supplementary Material

SC-OLF-D4SC08098J-s001
