# Supplementary material for: Deciphering the mechanism of hydrogen peroxide formation in ultrasound-mediated water-in-oil microdroplets
Source: Chem Sci. 2025 Mar 6;16(15):6450–7. doi: 10.1039/d4sc08098j (PMC11912498; doi:10.1039/d4sc08098j)
Supplement: SC-016-D4SC08098J-s001 [file SC-016-D4SC08098J-s001.pdf]

Supplementary Information (SI) for Chemical Science.
This journal is © The Royal Society of Chemistry 2025

Supporting Information for

Deciphering the Mechanism of Hydrogen Peroxide Formation in Ultrasound Mediated Water-in-Oil Microdroplets

Xiaohu Zhou, [†*a] Shutong Du, [†a] Wenjuan Zhang [a] and Bo Zheng [*a]

[a] Institute of Chemical Biology, Shenzhen Bay Laboratory, Shenzhen, 518132 China.

Email: zhouxh@szbl.ac.cn; bozheng@szbl.ac.cn

† X.Z. and S.D. contributed equally.

Experimental Procedures

Materials

n-hexadecane (n-16, 99%) was purchased from Thermo Fisher Scientific and used without further purification. Terephthalic acid (TA, 98%), acrylamide (99%), ammonium persulfate (APS, 98%) and sorbitan monooleate (Span 80, for GC) were purchased from Sigma-Aldrich. Potassium iodine (KI, 99%), 2-hydroxyterephthalic acid (hTA, 98%), 4-Carboxyphenylboronic acid (CPBA, 97%) and heavy-oxygen water ($H_2{}^{18}O$, 97 atoms % ^{18}O) were purchased from Macklin. Ammonium molybdate tetrahydrate ($(NH_4)_6Mo_7O_{24}\cdot 4H_2O$, 99.9%), sodium dihydngen phoshate anhydrous (NaH_2PO_4, 99%), nitrotetrazolium blue chloride (NBT, 98%), sodium chloride (NaCl, 99.5%), sodium sulfate anhydrous (Na_2SO_4, 99%), Tert-butyl alcohol (t-BuOH, 99.5%) and p-benzoquinone (p-BQ, 99%) were purchased from Aladdin. Hydrogen peroxide (H_2O_2, 30%) and hydrochloric acid (HCl, 36%) were purchased from Sinopharm. Sodium hydroxide (NaOH, 99%) was purchased from Boer. 5,5-dimethyl-1-pyrroline-N-oxide (DMPO) and 5-tert-butoxycarbonyl 5-methyl-1-pyrroline N-oxide (BMPO) were purchased from GLPBIO. Deuterium oxide (D_2O, 99.9%) was purchased from 3ABIO/Energy Chemical. All solutions were prepared using ultrapure water (18MΩ·cm^{-1}).

Generation of Microdroplets and H_2O_2

200 μL H_2O and 2 mL n-16 were added into a 10 mL brown vial. The reaction vial was placed above the ultrasonic transducer at the bottom of the ultrasonic water bath (SB-5200D, Scientz). The water level (20 mm) and temperature (24-28 °C) of the ultrasonic water bath were stable via continuous water circulation. The reaction sample was subjected to ultrasonication for the desired reaction time to generate microdroplets and H_2O_2. After ultrasound, in order to prevent microdroplet merger, the reaction vial was frozen with liquid nitrogen immediately, and part of the frozen emulsion was scraped out with a medicine spoon, observed by optical microscope (ECLIPSE Ni-U, Nikon) with camera, and analyzed by

the software ImageJ to obtain the size of produced microdroplets.

To investigate the effect of dissolved O_2, the reaction sample for ultrasound was placed in air and N_2 atmospheres, respectively. For the sample in N_2, the water-oil mixture was purged by N_2 for 15 min in the vial, and the vial lid was replaced by a N_2 balloon during ultrasound.

Quantification of Generated H_2O_2

After ultrasound, the aqueous solution was collected by centrifugation (15,000 rpm for 10 min, 5425R, Eppendorf), and then the H_2O_2 concentration was quantified via spectroscopic method. Two solutions were prepared in advance, namely a solution containing 0.4 M KI, 0.1 M NaOH, and 0.02 mM $(NH_4)_6Mo_7O_{24}\cdot 4H_2O$, and a solution containing 0.2 M NaH_2PO_4. The diluted sample solution was mixed with these two solutions at 1:1:1, and after 30 min for color development, the mixed solution was taken out for UV-Vis absorbance test (NanoDrop One^c, Thermo Scientific). The absorbance at 353 nm was taken as the characteristic value of I_3^-, and the standard curve was drawn according to the H_2O_2 solutions of known concentration below 500 μM.

Acrylamide polymerization test

Aliquots of aqueous phase solutions were collected from the bulk mixture of 200 μL 0.8 M acrylamide and 2 mL n-16 after ultrasonic treatment for 1 hour. Each aliquot had the volume of 450 μL. 50 μL D_2O was added to the aliquot solution, and the mixture was added into an NMR tube. ^1H NMR measurements were performed on NMR Spectrometer (Avance Neo 600MHz, Bruker). Control experiments included a 0.8 M acrylamide solution and a polyacrylamide (PAAm) solution polymerized from a 0.8 M monomer concentration using 10 mg mL^{-1} APS as an initiator at 80 ℃ for 5 minutes.

Reactive oxygen species (ROS) analysis

The ROS generated during the generation of H_2O_2 in microdroplets were detected by EPR measurement (EMX plus-6/1, Bruker) and active species quenching experiment. 200 μL 100 mM DMPO as aqueous phase and 200 μL 100 mM BMPO as aqueous phase are employed to detect hydroxyl radicals (·OH) and superoxide radicals ($\cdot O_2^-$), respectively. Radical quenching experiments were conducted by adding the sacrificial agents of t-BuOH (·OH), p-BQ ($\cdot O_2^-$), which were added before the reaction.

The quantitative determination of ·OH was based on the principle that TA itself does not fluoresce, however, when TA reacts with ·OH, the product hTA does fluoresce. The sample consisted of 200 μL 5 mM TA and 20 mM NaOH aqueous solution and 2 mL n-16. After desired irradiation time of ultrasound, aqueous solution was collected and diluted, and the fluorescence intensity was measured with an excitation/emission wavelength of 315/425 nm (SH1M-SN, Agilent). The standard curve was drawn according to the hTA solutions of known

concentration below 500 nM.

The quantitative determination of $\cdot O_2^-$ was based on the principle that NBT is water-soluble, however, when NBT reacts with $\cdot O_2^-$, the product monoformazan is insoluble and precipitates as blue solid, thus reducing the concentration of NBT in the solution. The sample consisted of 200 μL 3mM NBT aqueous solution and 2 mL n-16. After desired irradiation time of ultrasound, aliquot was collected and diluted for UV-Vis absorbance measurement at 259 nm to measure the concentration of NBT.

Oxygen Isotope Labeled Water and Dioxygen Experiment

The experiment of ^{18}O labeled water was done with 200 μL 20% $H_2{}^{18}O$ and 2 mL n-16. The ^{18}O labeled oxygen test sample consisted of 200 μL H_2O and 2 mL n-16. After the sample was bubbling through N_2 for 15 min, an $^{18}O_2$ balloon was placed on the bottle. After 10 min of ultrasound, 100 μL of the final aqueous phase solution was added to 400 μL 1 mM CPBA. The product 4-hydroxybenzoic acid was analyzed by mass spectrometer (Q Exactive Plus, Thermo Scientific).

Mass spectrometry analysis of $C_nH_nO_n$ products

We acquired electrospray ionization (ESI) full mass spectra (50.0000-250.0000 m/z) of ultrapure water and the water phase separated after 1 hour of ultrasound treatment of the emulsion using a Q Exactive Plus mass spectrometer (Thermo Scientific). $C_nH_nO_n$ products were preliminarily characterized through comparative analysis of differential peaks in the two mass spectra. Possible elemental compositions were calculated using Xcalibur software with a mass tolerance of under 10 ppm.

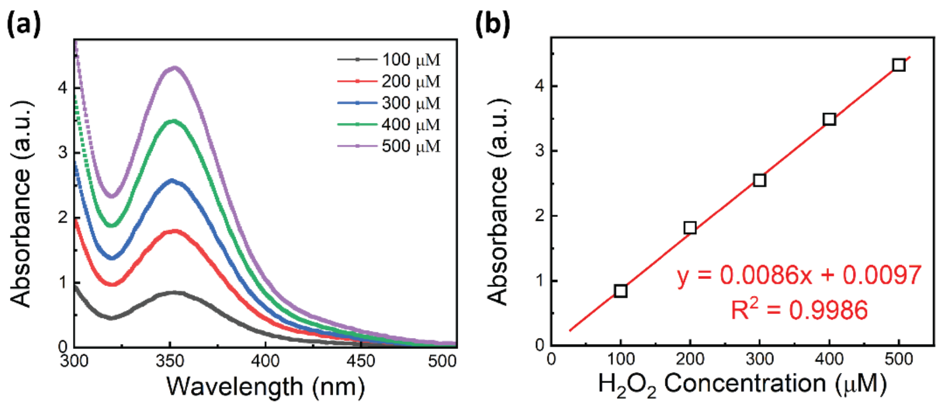

Figure S1 (a) The absorbance spectra of I_3^- according to the H_2O_2 concentrations from 100 μM to 500 μM. (b) Typical calibration curve for H_2O_2 concentration according on the absorbance peak at 353 nm. Higher concentrations above 500 μM were measured after dilution.

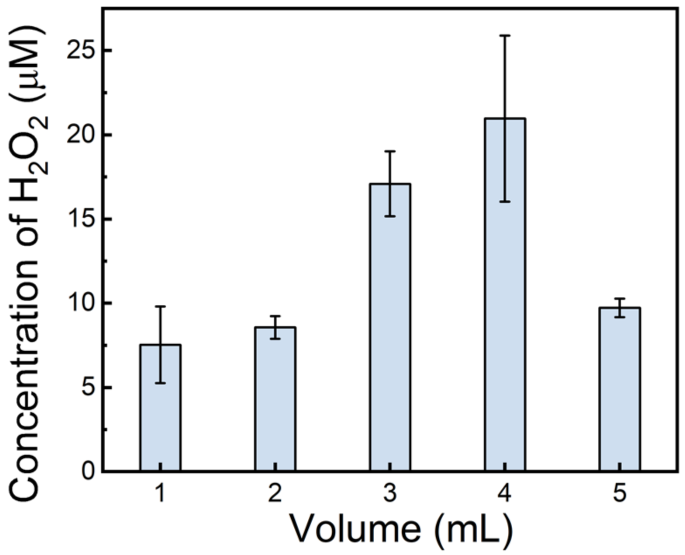

Figure S2 Quantification of H_2O_2 in single-phase bulk water with varying volumes under ultrasound irradiation within 5 minutes.

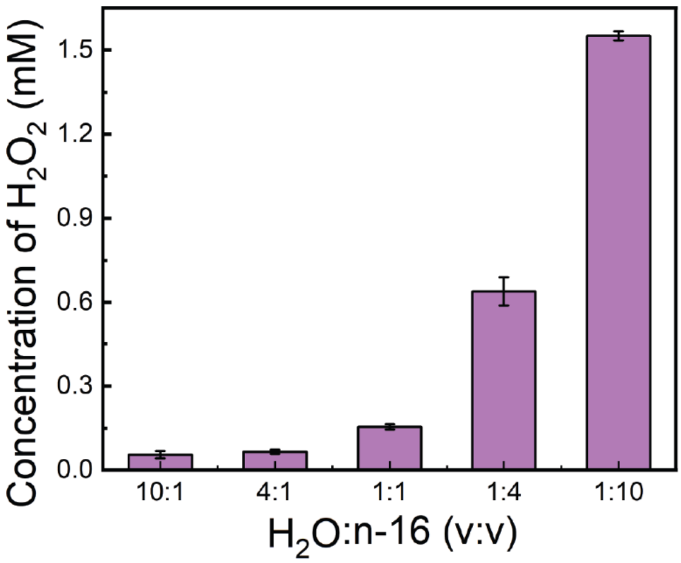

Figure S3 Quantification of H_2O_2 in water microdroplets under with varying water-to-hexadecane ratios under ultrasound irradiation within 5 minutes. Note that all samples maintained a constant total volume of 2.2 mL.

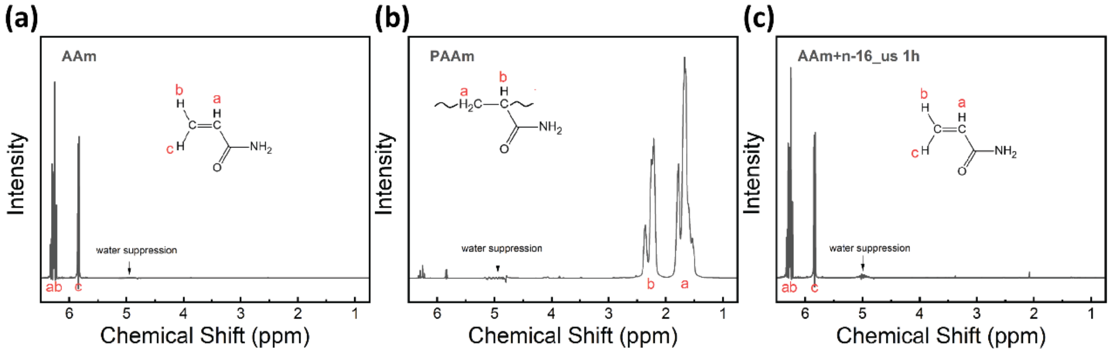

Figure S4 ^{1}H NMR spectral comparison of acrylamide polymerization: (a) acrylamide (AAm) monomer solution, (b) polyacrylamide (PAAm) solution, and (c) aqueous phase from water-in-oil microdroplets containing AAm monomers after 1 hour of ultrasound irradiation. The absence of PAAm signals in spectrum (c) indicates that no radical polymerization occurred in the water-in-oil microdroplets during ultrasound treatment.

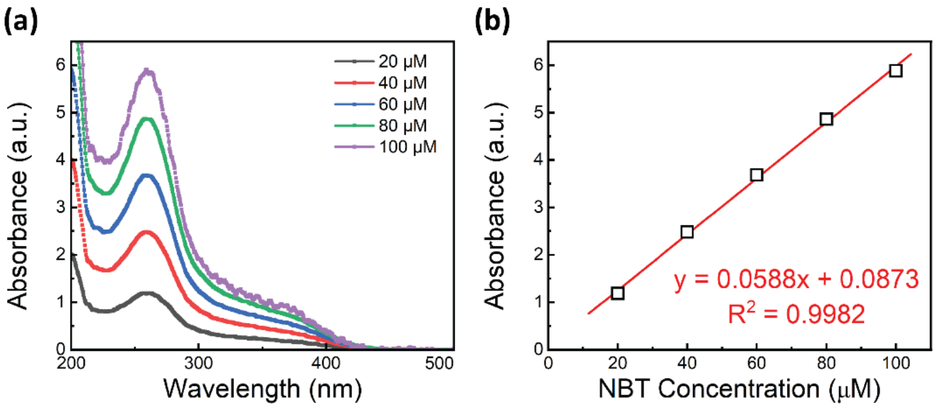

Figure S5 (a) The absorbance spectra of NBT from 20 μM to 100 μM. (b) Typical calibration curve according on the absorbance peak at 259 nm. Higher concentrations above 100 μM were measured after dilution.

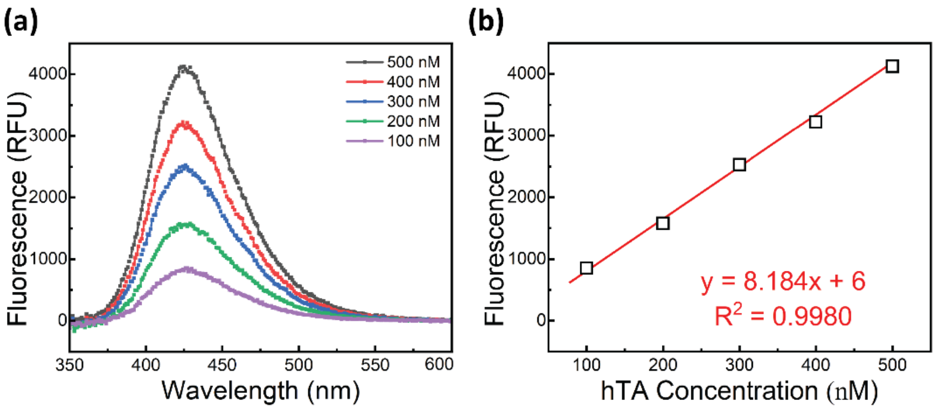

Figure S6 (a) Fluorescence emission spectra (excited at 315 nm) of hTA standards from 100 nM to 500 nM. (b) Typical calibration curve for hTA quantification using fluorescence spectrometry, and the intensity refers to the peak emission wavelength at 425 nm. Higher concentrations above 500 nM were measured after dilution.

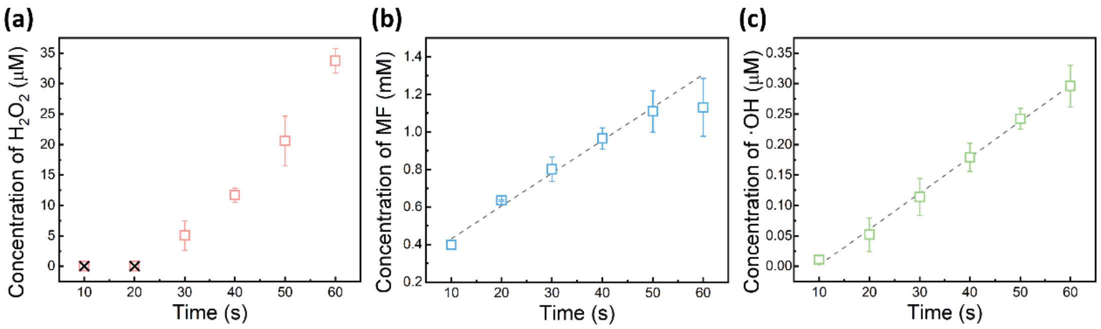

Figure S7 Quantification of (a) H_2O_2, (b) monoformazan (MF), and (c) ·OH in water microdroplets under ultrasound irradiation within 60 seconds after removal of the dissolved O_2 by N_2 purging.

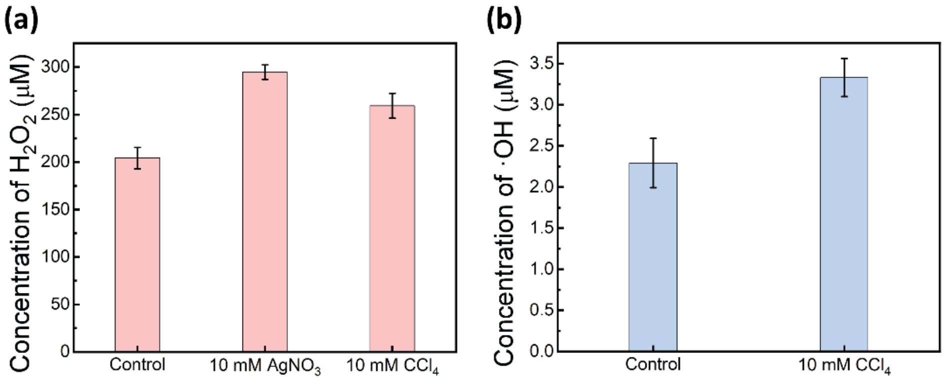

Figure S8 (a) Evolution of H_2O_2 in the presence of electron scavengers in the aqueous phase (10 mM $AgNO_3$) or oil phase (10 mM CCl_4) under 60 seconds ultrasound irradiation. (b) Evolution of ·OH radicals with 10 mM CCl_4 as an electron scavenger under 60 seconds ultrasound irradiation.

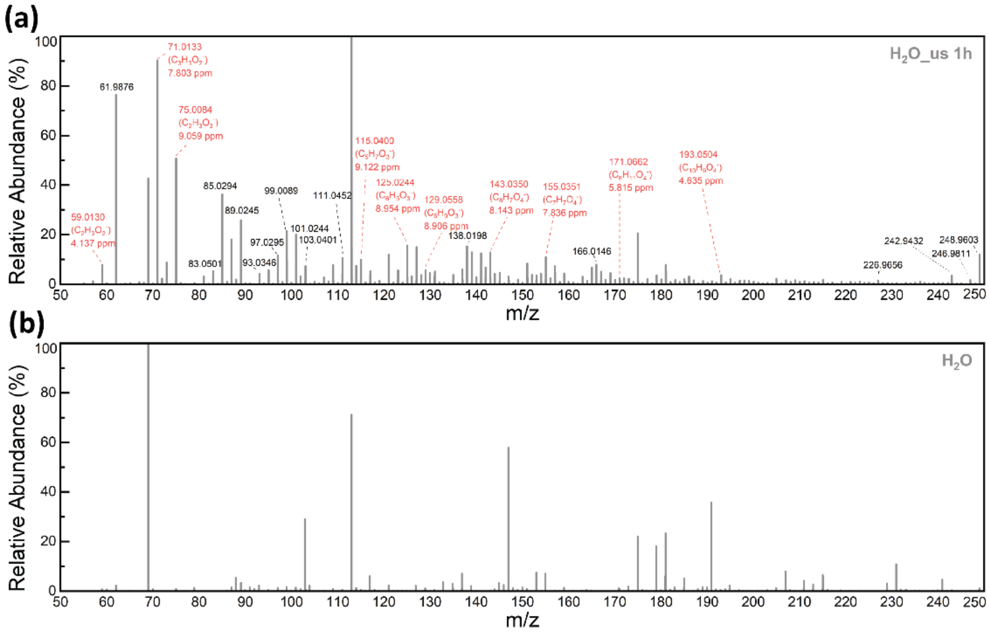

Figure S9 ESI-MS spectra of (a) ultrapure water and (b) the aqueous phase after 1 hour of ultrasound treatment of the water-in-hexadecane emulsion. The presence of new peaks in spectrum (b) demonstrates the formation of various $C_nH_nO_n$ compounds, indicating the reaction of ·OH radicals with hexadecane.
